# Obstructed or Malpositioned Urethral Catheter Induced Acute Kidney Injury

**DOI:** 10.1155/2012/731502

**Published:** 2012-10-11

**Authors:** Ankita Patel, Eli A. Friedman

**Affiliations:** Department of Medicine, SUNY Downstate Medical Center, 450 Clarkson Avenue, Brooklyn, NY 11203, USA

## Abstract

Unanticipated renal failure may be induced by an obstructed urethral catheter that was a component of complex management or difficult insertion. Two patients with new-onset uremia due to obstructed urethral catheters evinced rapid return of renal function when their blocked catheters were replaced.

## 1. Introduction

Current terminology of acute kidney injury (AKI) incorporates a broad spectrum of clinical disorders including prerenal azotemia, intrinsic renal injury, and obstructive uropathy. AKI may be associated with substantial morbidity and mortality attributed to uremia necessitating a logical approach to its prevention and early recognition permitting urgent management. 

We encountered 2 recent cases of AKI associated with acutely diminished urine output solely induced by mechanical obstruction of a urethral catheter. Prompt recognition and removal of a blocked urethral catheter as evidenced by these 2 patients may resolve the etiology of unexplained azotemia especially in patients lacking any history of renal disease.

## 2. Case Presentations

### 2.1. Patient 1

A 52-year-old African Caribbean man, actively employed as a security guard, known to have hypertension for 10 years came to the emergency room complaining of multiple episodes of nonbloody, watery diarrhea, after returning from a visit to Haiti 5 days earlier. His blood pressure was 90/60 mmHg with a pulse rate of 110 /min. On initial evaluation he appeared severely dehydrated with dry oral mucosa and decreased skin turgor. Admission laboratory values included hemoglobin of 16 g/dL, blood urea nitrogen (BUN) of 87 mg/dL, serum creatinine of 9.8 mg/dL, serum bicarbonate of 6 meq/L, an anion gap of 15 meq/L, and a serum albumin of 4.6 g/dL. An indwelling urethral catheter was inserted yielding 15 mL of urine over 24 hours. Urinalysis noted a specific gravity of 1.025 and urine sodium of 5 meq/L. The initial clinical diagnosis was AKI secondary to dehydration attributed to profuse diarrhea later proven to be due to cholera infection. Despite 2 days of aggressive hydration with normal saline, the patient continued to be anuric with a serum creatinine concentration rising to 10.4 mg/dL. At that time, abdominal examination detected a grossly distended bladder prompting removal of the initial urethral catheter which was found to be clogged with amorphous debris. A replacement catheter yielded approximately 1000 mL of urine over the first half hour. The fresh catheter permitted a sustained urine output of 100 mL/hour of urine with an associated decrease in serum creatinine concentration to 1.5 mg/dL within one day, at which point the patient was discharged to his home. Because AKI resolved upon replacement of a debris-plugged urethral catheter, no further imaging studies were performed. The patient continued free of urinary complaints.

### 2.2. Patient 2

**Figure 1:**
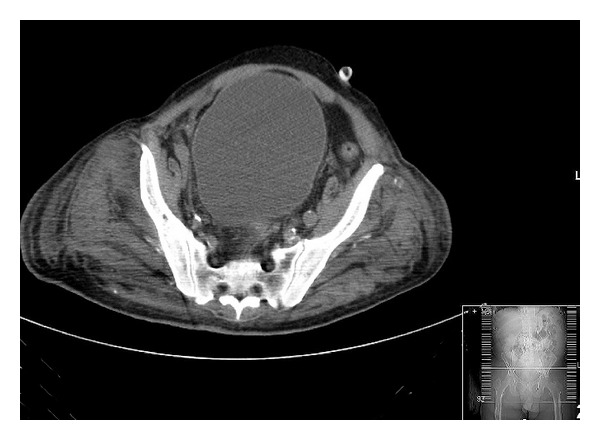
Patient 2: CT showing a grossly distended bladder blocking detailed visualization of the urethral catheter's location.

**Figure 2:**
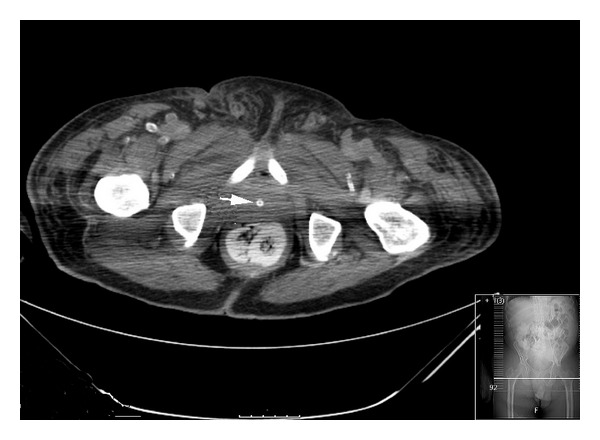
Patient 2: CT showing (arrow) the urethral catheter lodged in the prostatic urethra inducing obstruction to urinary flow.

A 73-year-old African American man, a bed-bound nursing home resident with dementia and hypertension of unknown duration, suffered a stroke attributed to a left middle cerebral artery occlusion necessitating hospitalization. Because of his immobility, a urethral catheter was inserted, yielding a urine output of approximately 10–15 mL per day for the first 2 days of hospitalization. By the third hospital day, his serum creatinine concentration had progressively increased from its level of 1.1 mg/dL on admission to 3.8 mg/dL. A repeat physical examination detected a grossly distended bladder which on a computed tomography scan of the abdomen was found to be associated with bilateral hydronephrosis and hydroureter, and a distended bladder with the catheter bulb lodged in the prostatic urethra inducing obstruction to urinary flow (Figures 1 and 2). Following insertion of a replacement urethral catheter, approximately 700 mL of urine drained immediately with a subsequent urine output of 50–70 mL/hour and a return of his creatinine level to 1.1 mg/dL over the next 24 hours.

Review of all available medical data prior to hospitalization in each of the two patients found no evidence of preexisting renal disease.

## 3. Discussion

As reported by Waikar et al., AKI affects about 15.3% of all hospitalized patients [1, 2]. Bagshaw et al. studied 5693 consecutive admissions and concluded that the presence and severity of AKI were independently associated with an increased mortality at 1 year [3]. In a study of 19,982 critically ill adults, an elevation of serum creatinine of greater than 0.5 mg/dL was associated with a 6.5-fold (95% confidence interval 5.0 to 8.5) increase in the risk of death, a 3.5-day increase in the length of hospital stay, and nearly $7,500 in excess hospital costs [4]. 

Acute urinary retention is a very common cause of AKI as currently defined. However, mechanical obstruction by a urethral catheter has been only rarely mentioned as a cause of urinary obstruction in standard medical texts. Our PubMed search conducted on July 18, 2012, using “obstructed urinary catheter” as keywords, returned a case report by El-Zoghby et al., of renal failure caused by obstruction due to a urethral catheter balloon malpositioned in the prostatic urethra [5]. An obstructed catheter was also noted to have caused transient acute urinary retention and life-threatening autonomic dysreflexia in a tetraplegic patient who did not have azotemia [6]. 

Our paper clearly illustrates that mechanical obstruction by urethral catheters may induce substantial morbidity. A malpositioned urethral catheter should be considered among possible etiologies in any patient with unexplained AKI, especially when associated with an acute reduction in urinary output that occurs or persists after urethral catheter insertion. Prompt intervention including replacement of the obstructed urethral catheter may, as in our two patients, lead to a rapid improvement in renal dysfunction. The need for expensive and time-consuming investigational modalities is thus avoided and the length of hospital stay is shortened once the catheter problem is recognized and resolved.

## 4. Summary

We describe two patients with AKI resulting from obstructive uropathy that rapidly resolved after replacement of their obstructed urethral catheters. These cases underscore the pragmatic reality that the in situ presence of a urethral catheter does not exclude the possibility that acute urinary obstruction is the cause of AKI. The differential diagnosis of unexplained AKI associated with reduced urine output, in patients with a urethral catheter, should include affirmation of both the proper position and patency of the catheter.
